# Supplementary material for: Simulating transport and distribution of marine macro-plastic in the Baltic Sea
Source: PLoS One. 2023 Jan 19;18(1):e0280644. doi: 10.1371/journal.pone.0280644 (PMC9851513; doi:10.1371/journal.pone.0280644)
Supplement: S1 File — (PDF) [file pone.0280644.s001.pdf]

Supporting information

S1 Spatial scaling of Green's functions The Green's function G can be understood as the pollution plume originating from a particular pollution point source. To understand the complex spatial nature of Green's functions, it is of interest to study simplified models, where the Green's functions can be worked out analytically. The advection-diffusion-dissipation (ADD) transport equations needed to be solved to determine G have 3 essential parameters:

- The advection field $v(x,t)$, which includes surface currents, wind drag and Stokes drift all together.
- The horizontal diffusivity $D_h(x,t)$, representing sub grid scale eddies as a stochastic random walk process.
- The local sinking (or removal) rate $\lambda(x,t)$

In the analytical exploration below, these are all considered spatially and temporally constant, i.e. $v(x,t) = v$, $D_h(x,t) = D_h$ and $\lambda(x,t) = \lambda$.

The first simple reference system is the 1D case in the no advection limit $v = 0$, so x is scalar. We arbitrarily place the source with an influx of S units per time at $x = 0$. At stationarity we get

$$\frac{\partial G}{\partial t} = D_h \frac{\partial^2 G}{\partial x^2} - \lambda G = 0 \tag{13}$$

$$S = -D_h \frac{\partial G}{\partial x}\Big|_{0_-}^{0_+} \tag{14}$$

which is solved by $G = A\exp(-kx)$ with $k_\pm = \pm\sqrt{\lambda/D_h}$ and $B = S/\sqrt{\lambda D_h}$, if the problem is confined to $x > 0$. If free boundary conditions apply to the right, only the k_+ branch applies.

Second we consider downstream advection $v > 0$ and get

$$\frac{\partial G}{\partial t} = -\frac{\partial}{\partial x}\left(-D_h \frac{\partial G}{\partial x} + vG\right) - \lambda G = 0 \tag{15}$$

$$S = \left(-D_h \frac{\partial G}{\partial x} + vG\right)\Big|_{0_-}^{0_+} \tag{16}$$

and we again find exponential solutions with

$$k_{\pm} = -\frac{v}{2D_h} \pm \sqrt{(\frac{v}{2D_h})^2 + \frac{\lambda}{D_h}} \tag{17}$$

for the downstream side ($x > 0$), giving again $k_{\pm} = \sqrt{\lambda/D_h}$ in the diffusive limit ($v \to 0$), and $k = \frac{\lambda}{v}$ in the advective limit ($D_h \to 0$). . If free boundary conditions apply to the right, only the k_+ branch applies in the diffusive limit. If the problem is confined to $x > 0$, this implies $B = S/(v + D_h k_+)$. If the problem is solved with specific right side boundary conditions at $x = L$, e.g. $G(L) = P_0$, the solution is a linear combination of branches $k_{\pm}$ satisfying flux boundary conditions to the left $x = 0$ and the specified boundary condition to the right ($x = L$). The limiting solutions suggest to consider the dimensionless number

$$\chi = \frac{v^2}{D_h \lambda} \tag{18}$$

which tells whether the pollution plume tail is advection like ($\chi >> 1$) or diffusion like ($\chi << 1$). In this way, χ corresponds to the Péclet number for the advection-diffusion problem, telling whether transport on a given scale is advection or diffusion like. An alternative way to identify that χ characterizes the dynamical regime is that χ and $1/\chi$ gives the leading correction to k in the diffusion and advection limit, respectively.

Exploring dimensionality, we may consider the 2D diffusion-dissipation problem at stationarity with the source at $r = 0$:

$$(D_h \nabla^2 - \lambda) G = 0 \tag{19}$$

where the regular solution is

$$G \sim K_0(kr) \tag{20}$$

again with $k = \sqrt{\lambda/D_h}$ as for 1D, and

$$K_0(z) \sim 1.25 \frac{\exp(-z)}{\sqrt{z}} \tag{21}$$

being the modified Bessel's function of second kind and 0th order.

S2 Back diffusion correction In the derivation of the DRRS scheme, we included only advective contributions along the open (water bound) boundaries of the system. If advection locally were into the system that was handled as a local plastic point source. If the Lagrangian ratio

$$\xi = \frac{1}{\sqrt{2}} \frac{v\ dt}{\sqrt{2D_h dt}} = \frac{v}{2}\sqrt{\frac{dt}{D_h}} \tag{22}$$

is small, diffusive transport is locally dominating, for a given particle time step dt. This means that a small number of particles will diffuse back into the system, if the boundary concentration $P_0 > 0$, even if the advective direction is out of the system, for small ξ. For the 1D case, it is follows from Eq. 3 (using normal variates for η for analytical convenience) that the number of back diffusing particles in a time step dt is

$$n_b = P_0 \sqrt{2D_h dt}\ g(\xi) \tag{23}$$

$$g(\xi) = \frac{1}{\sqrt{2\pi}} \exp(-\xi^2) - \frac{1}{\sqrt{2}} Erfc(\xi) \tag{24}$$

If $P_0 >> 0$ and $\xi << 1$ point sources along the open boundaries corresponding to may be applied in the DRRS scheme. Backdiffusing particles should be distributed according to

$$p(z < 0) \sim Erfc(\frac{v\ dt - z}{\sqrt{2D_h dt}}) \tag{25}$$

away from the boundary normal, with $z = 0$ corresponding to the boundary, and $z < 0$ the system interior, if Gaussian statistics is applied for η. Sampling from distribution Eq. 25 is not commonly supported in numerical libraries, but n variates from Eq. 25 are efficiently generated by solving $C(U(0,1,n)) = x)$ with a few vectorized Newton-Raphson steps, where C is the cumulative density of Eq. 25, and $U(0,1,n)$ a random variate of length n on $]0,1]$. Corresponding equations can be derived for 1D/2D boundaries, corresponding to positively buoyant particles or microplastic dispersed vertically. From a technical perspective, neglecting the Lagrangian back diffusion correction strictly corresponds to solving the Eulerian problem Eq. 15 with boundary condition $P_0 = 0$ to the right side. The back diffusion correction, when $P_0 >> 0$ at the boundary is usually small and may be neglected in many real cases; in our paper case study we did not include the back diffusion correction, corresponding to assuming $P_0 = 0$.

S3 Benchmarking the DRRS algorithm In this section the advantages of the DRRS algorithm over simple non-resampling Lagrangian algorithms are demonstrated for the simple reference system described above, where the mathematical solutions can be resolved analytically. In Figure 11 we compare the performance of the DRRS

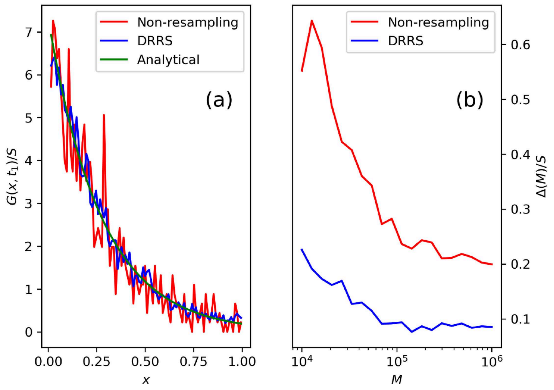

Fig 11. Comparison of Lagrangian schemes for resolving plastic concentration for a point source. (a) Typical plastic downstream concentration profiles as obtained by Lagrangian simulations and grid statistic, using either a simple non-resampling or the DRRS scheme, and compared to the analytical solution, with number of particles $M = 10^4$ in both cases. The plastic source is at $x = 0$ and the simulation domain is $0 < x < 1$. Water current v is from left to right. (b) The convergence of the standard deviation Δ_M as function of number of particles M, when compared to the analytical solution (figure (a), green curve) for non-resampling and DRRS Lagrangian schemes, respectively.

algorithm over the simple non-resampling Lagrangian. We let source emission $s = 2\ 10^6$ plastic items per time unit, and define domain size $L = 1$, setting the overall length scale of the problem, $\lambda = 0.5$ per time unit, $v = 0.1L$ per time unit, and $D_h 0.01L^2$ per time unit. To allow the system to equilibrate fully for both algorithms, the simulated period is $t_{sim} = 2\ max(1/v, 1/\lambda)$, starting from a homogeneous distribution $G(x) = s/(\lambda L)$ plastic items per length, which corresponds to the equilibrium concentration for the isolated system (without advection-diffusion loss). The number of particles in the simulation is $M = 10^4$. To convert the particle distribution into a concentration variable $G(x)$, the domain $0 < x < L$ was divided into 100 cells of width $\Delta x = 0.01L$, and number of particles in each cell n_i was counted and associated with the mid point, i.e. $G(x_i) = n_i R/\Delta x$, where R is the number of plastic items per particle. For the non-resampling algorithm, $R = s(1/\lambda + t_{sim})/M$ making optimal usage of the particle capacity, whereas for the DRRS R was determined from Eq. 9. In both runs the Lagrangian time step was $dt = 0.1$ and the smoothing time scale $T = 3dt$ for the DRRS run.

Figure 11(a) show the typical plastic concentrations $G_M(x)$ at the end of the simulation $t = t_{sim}$. The green line shows the analytical solution Eq. 17, toward which both algorithms converge for $M \to \infty$ The oscillations on the curves reflect the sampling uncertainty due to finite number of particles M in the simulations. For same number of particles we see that the DRRS algorithm resolve the plastic concentrations $G(x)$ better than the straightforward non-resampling algorithm, i.e. has smaller

oscillations around the correct asymptotic value (green line). Figure 11(b) show the convergence of the standard deviation $\Delta_{MT} = \sqrt{< (G_{MT}(x) - G(x))^2 >}$ for both algorithms (T not applicable for non-resampling scheme, where R is fixed); this plot shows that the standard deviation Δ_{MT} converges much faster for the DRRS algorithm, as heralded previously, so that the same accuracy can be achieved with fewer particles in the simulation (i.e. the simulation is computationally faster), or a more accurate result can be achieved with the same calculational load. The reason for the efficiency gain is that all particles in the simulation are active throughout the simulation in DRRS, whereas some must be reserved in the non-resampling algorithms so that continuous release of plastic at $x = 0$ can be simulated (in our benchmark all particles in the non-resampling Lagrangian run were active at some point in the simulation).

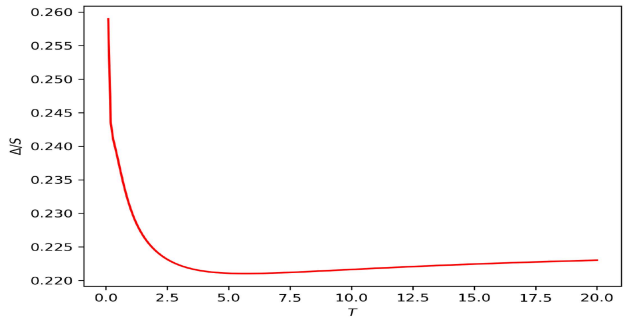

Fig 12. The deviation Δ_{MT} of the plastic concentration $G_{MT}(x)$ from the analytical solution $G(x)$ for the DRRS scheme, depending on the smoothing time scale T. In this plot $M = 10^4$.

Figure 12 show the deviation Δ_{MT} of the plastic concentration $G_{MT}(x)$ for the DRRS scheme, depending on the smoothing time scale T. As anticipated, the sensitivity with T on the deviation Δ_{MT} is small, if $T >> dt$; for real cases T should not be chosen extremely large, as the value of T becomes the lower time resolution limit for trends $G_{MT}(x,t)$, when forcing parameters (s, λ, v, D_h) are slowly varying. The Python code implementing the 1D simple reference system, along with Lagrangian vectorized resampling and non-resampling algorithms, are available as described below.

S4 Software availability The computer code behind the reported work is made openly available under LGPL license at the github.com repository.

S4.1 Realistic Lagrangian simulation tool The DRRS algorithm has been implemented within the IBMlib framework [43] as a task module, and demonstrated as reported in the present work. The setup allows to couple with realistic physical forcings (i.e. topography, hydrography, wave and wind fields), which must be established independently before conducting actual simulations; the particle state module allows to include various aspects of floating plastic e.g. biofouling and parametric variability representing different fractions. Building an IBMlib executable requires a Fortran 90+

compiler, gmake, Python3 and possibly netCDF support, depending on I/O formats. The code compiles on Linux/Unix platforms as distributed and may be cross-compiled to Windows platforms upon request. The code can be downloaded at https://github.com/IBMlib/IBMlib The suggested starting template for DRRS simulations is (file config.mk)

```
PARTICLE_STATE_DIR  = $(IBMLIB_DIR)/biology_providers/plastic
TASK_DIR            = $(IBMLIB_DIR)/task_providers/DRRS
```

Additionally, a physics API needs to be selected/configured to provide access to a local database of physical forcings (i.e. topography, hydrography, wave and wind fields). Actual physical forcing databases not distributed with the IBMlib framework.

S4.2 1D DRRS demonstration sandbox The Lagrangian simulation demonstration of the simplest spatial reference system of a pollution plume analyzed in S1 Spatial scaling of Green's functions, S2 Back diffusion correction and S3 Benchmarking the DRRS algorithm. The object-oriented demonstration is implemented in Python3, using NumPy and SciPy, and dynamics vectorized for optimal performance. The demonstration module is available at https://github.com/IBMlib/IBMlib/tree/master/sandbox/DRRS

S4.3 Configuration for the Baltic case study The IBMlib configuration and input files for the Baltic case study are provided in https://github.com/IBMlib/IBMlib/tree/master/task_providers/DRRS/Baltic_case_study. This includes scripts that setup baseline simulations (Greens functions and regional plastic distribution dynamics) with parameterization as described in this paper. This also includes the plastic source map input file and a README file explaining the setup and how to obtain physical forcing data. The installation and requirements of IBMlib are described in https://github.com/IBMlib/IBMlib/tree/master/doc .
