# Supplementary material for: Simulating transport and distribution of marine macro-plastic in the Baltic Sea
Source: PLoS One. 2023 Jan 19;18(1):e0280644. doi: 10.1371/journal.pone.0280644 (PMC9851513; doi:10.1371/journal.pone.0280644)
Supplement: Attachment — Submitted filename: Response_to_Reviewers.pdf [file pone.0280644.s002.pdf]

Adressing journal requirements

Dear Editor,

please find below our point-by-point response (green color) to journal requirement amendments (red color) raised to manuscript PONE-D-22-21796 Simulating transport and distribution of marine macroplastic in the Baltic Sea.

1. Please ensure that your manuscript meets PLOS ONE's style requirements, including those for file naming.

We believe that the resubmitted manuscript adheres to PLOS ONE's style requirements. This has been ensured by applying the provided latex template (plos_latex_template.tex) and bibliographic style (plos2015.bst) and manual checking of the manuscript PDF rendering.

2. Thank you for stating in your Funding Statement:
"This work was supported ... by this grant."

Please provide an amended statement that declares *all* the funding or sources of support (whether external or internal to your organization) received during this study, as detailed online in our guide for authors at http://journals.plos.org/plosone/s/submit-now. Please also include the statement “There was no additional external funding received for this study.” in your updated Funding Statement.
Please include your amended Funding Statement within your cover letter. We will change the online submission form on your behalf.

We have extended the funding statement as follows (changes in italics):

This work was supported by the European Commission (https://ec.europa.eu) under the Horizon2020 programme as project CLAIM (Cleaning Litter by developing and Applying Innovative Methods in european seas), grant agreement No. 774586. The contribution of all authors (AC,JM,JS,MstJ) were *fully* funded by this grant. *We acknowledge the support of the Technical University of Denmark, in providing the computational facilities used for transport simulations. There was no additional external funding received for this study. The funders had no role in study design, data collection and analysis, decision to publish, or preparation of the manuscript.*

3. We note that Figures 1, 4, 5, 6, 7 and 8 in your submission contain map/satellite images which may be copyrighted. All PLOS content is published under the Creative Commons Attribution License (CC BY 4.0), which means that the manuscript, images, and Supporting Information files will be freely available online, and any third party is permitted to access, download, copy, distribute, and use these materials in any way, even commercially, with proper attribution. For these reasons, we cannot publish previously copyrighted maps or satellite images created using proprietary data, such as Google software (Google Maps, Street View, and Earth). For more information, see our copyright guidelines: http://journals.plos.org/plosone/s/licenses-and-copyright.

We require you to either (1) present written permission from the copyright holder to publish these figures specifically under the CC BY 4.0 license, or (2) remove the figures from your submission:

a. You may seek permission from the original copyright holder of Figures 1, 4, 5, 6, 7 and 8 to publish the content specifically under the CC BY 4.0 license.

We recommend that you contact the original copyright holder with the Content Permission Form (http://journals.plos.org/plosone/s/file?id=7c09/content-permission-form.pdf) and the following text: “I request permission for the open-access journal PLOS ONE to publish XXX under the Creative Commons Attribution License (CCAL) CC BY 4.0 (http://creativecommons.org/licenses/by/4.0/). Please be aware that this license allows unrestricted use and distribution, even commercially, by third parties. Please reply and provide explicit written permission to publish XXX under a CC BY license and complete the attached form.”

Please upload the completed Content Permission Form or other proof of granted permissions as an ""Other"" file with your submission.

In the figure caption of the copyrighted figure, please include the following text: “Reprinted from [ref] under a CC BY license, with permission from [name of publisher], original copyright [original copyright year].”

b. If you are unable to obtain permission from the original copyright holder to publish these figures under the CC BY 4.0 license or if the copyright holder’s requirements are incompatible with the CC BY 4.0 license, please either i) remove the figure or ii) supply a replacement figure that complies with the CC BY 4.0 license. Please check copyright information on all replacement figures and update the figure caption with source information. If applicable, please specify in the figure caption text when a figure is similar but not identical to the original image and is therefore for illustrative purposes only. The following resources for replacing copyrighted map figures may be helpful:

USGS National Map Viewer (public domain): http://viewer.nationalmap.gov/viewer/
The Gateway to Astronaut Photography of Earth (public domain):
http://eol.jsc.nasa.gov/sseop/clickmap/

Maps at the CIA (public domain):
https://www.cia.gov/library/publications/the-world-factbook/index.html and
https://www.cia.gov/library/publications/cia-maps-publications/index.html
NASA Earth Observatory (public domain): http://earthobservatory.nasa.gov/
Landsat: http://landsat.visibleearth.nasa.gov/
USGS EROS (Earth Resources Observatory and Science (EROS) Center) (public domain):
http://eros.usgs.gov/#
Natural Earth (public domain): http://www.naturalearthdata.com/

All subfigures in figures 1,4,5,6,7 and 8 were generated using the python Matplotlib Basemap Toolkit package. The package is open source, distributed at GitHub (https://github.com) under an open source license (https://github.com/matplotlib/basemap/blob/develop/LICENSE). For the results(output) of the software program, all rights belong to the user, as stated in the license. Therefore the above mentioned figures may be published under CC BY 4.0 license by PLOS ONE without copyright infringement.

Response to reviewer 1

Please find below our point-by-point response to reviewer 1; reviewer comments in red, our response and modifications in green. Trivial typographical remarks have been fixed without summary below. For easier reference, all line/figure numbering refers to the initial submission.

no major issues were found from my side. Just a biologist's note at Line 469: crustaceans are only a minor component of biofouling communities, indeed most of the buoyancy changes are very likely due to intensive fouling by macro and micro-algae, not crustaceans. I suggest to remove or change this.

The sentence:

It is believed to be a dynamic rate, depending significantly on local physical conditions, planktonic dynamics, and abundance of crustacean settlers accumulated over the drift time of the plastic item.

was replaced with this:

It is believed to be a dynamic rate, depending significantly on local physical conditions and the abundance of macro and micro-algae and other potential settling organisms like bivalves, accumulated over the drift time of the plastic item.

(I have seen several pictures of marine macro-plastic with bivalves attached).

Response to reviewer 2

Please find below our point-by-point response to reviewer 2; reviewer comments in red, our response and modifications in green. Trivial typographical remarks have been fixed without summary below. For easier reference, all line/figure numbering refers to the initial submission.

3. Have the authors made all data underlying the findings in their manuscript fully available?
Reviewer #2: No

Unfortunately, the reviewer is not specific about what this exactly concerns. In a general sense, the data underlying our paper is constituted by code, input data and output data.

- *Code*: in Supporting information (section "S4 Software availability") we have in detail accounted for the full and unrestricted access to the code underlying our reported findings. Our code is distributed as open source at https://github.com/IBMlib/IBMlib .
- *Input data:* physical forcing data applied in our study has been described fully in section "Materials and methods"; we do not have distribution rights to the physical forcing data applied and can consequently not supply this, but in "Materials and methods" it is made clear what product is used and who the producer is, so they can be contacted by independent investigators. Some of the forcing data (wind fields and Stokes drift) is open access, whereas some restricted access (current fields). The plastic source mapping is available and that has now been emphasized in section "S4 Software availability". All scripts for for baseline runs (Greens functions and regional plastic distribution dynamics) are also now made available, and these scripts generate input files for running simulations with parameter setting as described in "Materials and methods" and Table 1. A README file is provided in a DDRS software folder for the configuration applied in the current paper. All this has now been emphasized in section "S4 Software availability" in a new paragraph "S4.3 Configuration for the Baltic case study".
- *Output data:* these high resolution binary gridded data sets containing two space and one time dimension are very large and unsuitable for redistribution. It is not customary that such extensive binary data sets are published as part of a scientific publication. Since our code and input data are fully described or provided, as outlined above, any independent investigator may recreate the output data, if needed.

Consequently, we argue that our reported findings are strictly reproducible by anybody that wants to reproduce our findings or extend our work.

I have some doubts about the methods. The hydrodynamic modelling plan is not clear. Parameters and detailed modelling processing used in this study are suggested. Would you describe the process of this simulation experiment in detail? Add more specifications of the study area, and include a new subsection.

A new subsection has been added to "Materials and methods" that explicitly describes the baseline simulations (Greens functions and regional plastic distribution dynamics) that underlies our paper. The technical aspects of the simulations has already been described, as the reviewer acknowledges.
Key facts about the physical oceanography of the study area has already been provided in the first lines of "Materials and methods"; we have extended this with a few details about biological oceanography and geography, and distilled this out into a new subsection "Study area: the Baltic Sea" that should give the reader a more comprehensive impression of the study area.

What is the database used for the identification of the macro litter sources?

We have now inserted the exact reference for the macro litter source data set applied in this study; further we note that the macro litter source data set formatted to Lagrangian simulations is made available to the scientific community, as described in the new paragraph "S4.3 Configuration for the Baltic case study".

Explain better the assumption of the 4g/item (line 147).

This is not an assumption, but an observation that if w = 4 g/item then total riverine and coastline input are equal *in this data set*. We add *in this data set* to emphasize that this threshold value applies to that data set, and different threshold value must be expected for other data sets, and start the sentence with *We determine that ...* to make statement clearer. As stated in the next sentence we apply w = 10 g/item in our simulations, as supported by a reference, i.e. not w = 4 g/item (we added *in our simulations* to make this clearer).

Line 167-168: What is the uw? This parameter is not mentioned in the text.

---

u_w designate drift velocity; this is now indicated in line 165, before first occurrence iin Eq. 1

The section on the material methods is more confusing. I suggest authors try to be more concise.

Some confusion emerge from an inconsistent usage of headings "Materials and methods"; this is now amended. Now our "Materials and methods" consistent of these 8 sub sections:

```
\subsection*{Study area: the Baltic Sea}
\subsection*{Baltic Sea physical model}
\subsection*{Baltic Sea macro litter sources}
\subsection*{Baltic Sea macro litter transport model}
\subsection*{The DRRS scheme for quasi-equilibrium distributions}
\subsection*{Green's functions and plastic distribution}
\subsection*{Baseline simulations}
\subsection*{Model validation}
```

the latter two of which was suggested by the present reviewer. These sub sections represent a logical progression and the modularity of our approach, and should aid the reader to zoom in on aspects of interest. We hope the latest revisions meet to request of the reviewer, as this reviewer comment unfortunately is not very specific. We also draw attention to the fact that our paper present methodological progresses (a publication type warranted by the editorial guidelines), and therefore our section is slightly longer than papers reporting results obtained by standardized approaches.

The first paragraph of the results section should be on the methods (lines 351-362). Idem for lines 420-425. The results section must be restructured.

---

lines 351-362: we have followed the reviewers suggestion and moved the model validation paragraph to the "Materials and methods" section.

lines 420-425: Presentation style in natural sciences across journals and authors is quite diverse; the only apparent consensus in writing guides is that the premises of the results should be fully reported in "Materials and methods" (with possible links to supp. material), so that presented results are completely reproducible by independent investigators. The content of lines 420-429 is not methods used to *produce* material to the section "Results", but *reflections* on the patterns of material in the section "Results"; therefore we feel that this content belongs here. We have inlined the previous equation 12, so that it is more clear that it is part of our observations on the simulation output rather than part of the method description. We browsed the recent literature in different journals and quickly found 3 examples of papers with reflection equations is sections Results/Discussion:

Ecological Modelling 474 (2022) 110153 (page 4)
https://doi.org/10.1098/rstb.2020.0414
https://doi.org/10.1371/journal.pone.0278167 (result dsicussion)

However, if the editor agree with the reviewers comment on lines 420-425, we are willing to move this to the sub section "Green's functions and plastic distribution", even though we think it is slightly misplaced there and will make the "Materials and methods" section bulkier.

Further, to enhance the overview of our presented results, we have subdivided the "Results" section into 3 sub sections,

\subsection*{Macroplastic dynamics in the Baltic Sea}
\subsection*{Green's functions for pollution sources}
\subsection*{Transport mechanisms}

The authors only considered constant litter influx (line 538), may the authors should also discuss the contribution by other sources if considered. The authors should supply information on land use around the bay and discuss other pathways of plastic pollution.

The applied plastic sources maps are supposed to represent all land-based sources
In lines 519-537 we extensively discussed abandoned, lost and discarded fishing gear (ALDFG), another important recognized source of plastic pollution not included in the source map applied. We have extended this section with other sources including other nautical activity (e.g. shipping, ferrying and leisure), offshore platforms, aquaculture and abandoned vessels, added references for the ratio of land/sea-based pollution and suggested future amendments. Even though land use is an interesting subject, we find a digression on land use around the bay (Baltic Sea?) to be beyond the scope of our paper as only the net input is of importance for our study (not why) and we prefer readers interested in this topic consult the relevant literature.

Please insert the color bar percentage in Figure 1b.

Figure 1b is only a schematic view, reproduced from another source. The arrows represents large-scale water mass transport, and a scale-bar for volume transport is not available from the source. We have now noted in the figure caption that arrows represent a schematic view.

In figure 6 the color bars have to be adjusted so that the same color scale is used in all panels.

Fig. 6a compared to Fig. 6b,c,d show different things, and therefore it is not appropriate to apply same color scale. Fig 6a is absolute concentration, whereas Fig. 6b,c,d are concentration *relative* to the local long-term average concentration. We find this way of plotting more informative, as it better display contrasts. Value range of Fig. 6a compared to Fig. 6b,c,d is very different, and therefore applying same scale (even for a logplot) will remove much of the contrast in all figures. We have reformulated the caption so the difference between Fig. 6a and Fig. 6b,c,d should be clearer.
